# The Economic Cost of Diet and Its Association with Adherence to the Mediterranean Diet in a Cohort of Spanish Primary Schoolchildren

**DOI:** 10.3390/ijerph18031282

**Published:** 2021-01-31

**Authors:** Rosario Pastor, Noemi Pinilla, Josep A. Tur

**Affiliations:** 1 Research Group on Community Nutrition and Oxidative Stress, IUNICS, University of the Balearic Islands, E-07122 Palma de Mallorca, Spain; rosario.pastor@ucavila.es; 2 Faculty of Health Sciences, Catholic University of Avila, 05005 Avila, Spain; noemipini22@gmail.com; 3 CIBEROBN (Physiopathology of Obesity and Nutrition), Instituto de Salud Carlos III, 28029 Madrid, Spain; 4 Foundation Health Research Institute Balearic Islands (IDISBA), E-07120 Palma de Mallorca, Spain

**Keywords:** diet quality, Mediterranean diet, Mediterranean Adequacy Index, monetary cost

## Abstract

*Background*: Adoption of a certain dietary pattern is determined by different factors such as taste, cost, convenience, and nutritional value of food. *Objective*: To assess the association between the daily cost of a diet and its overall quality in a cohort of 6–12-year-old Spanish schoolchildren. *Methods*: A cross-sectional survey was conducted on a cohort (*n* = 130; 47% female) of 6–12-year-old children schooled in primary education in the central region of Spain. Three-day 24 h records were administered, and the nutritional quality of the diet was also determined by means of Mediterranean Adequacy Index (MAI). A questionnaire on sociodemographic data, frequency of eating in fast-food restaurants, and supplement intake were also recorded. The person responsible for the child’s diet and the schooler himself completed the questionnaires, and homemade measures were used to estimate the size of the portions. Food prices were obtained from the Household Consumption Database of the Spanish Ministry of Agriculture, Fisheries and Food. The economic cost of the diet was calculated by multiplying the amount in grams of the food consumed by each child by the corresponding price in grams and adding up the total amount for each participant. The total economic cost of the diet was calculated in €/day and in €/1000 kcal/day. *Results*: The area under the curve (AUC) for €/day and €/1000 kcal/day represent 62.6% and 65.6%, respectively. According to AUC values, adherence to Mediterranean diet (MD) is a moderate predictor of the monetary cost of the diet. A direct relationship between the cost of the diet and the adherence to MD was observed [OR (€/1000 kcal/day) = 3.012; CI (95%): 1.291; 7.026; *p* = 0.011]. *Conclusions*: In a cohort of Spanish schoolchildren with low adherence to the MD, a higher cost of the diet standardized to 1000 kcal was associated with above-average MAI values.

## 1. Introduction

An optimal diet (balanced, varied, healthy, personalized, palatable, and functional) has positive effects on physical and mental health [1,2]. The Mediterranean diet (MD) has been recognized as an optimal diet model, and scientific evidence has shown health benefits, especially in the prevention and control of noncommunicable diseases in adults, children, and adolescents [3,4,5,6,7]. Prior studies have shown that Spanish children and adolescents move away from the MD pattern [8].

To assess the quality of the diet, different indices have been used, such as the Diet Quality Index-International (DQI-I). However, this index may not be fully adapted to the MD pattern [9]. Others such as the Mediterranean Diet Score (MDS) or the Mediterranean Adequacy Index (MAI) have been also used [10,11,12].

Adoption of a certain dietary pattern is determined by different factors such as the taste, cost, convenience, and nutritional value of food [13]. Regarding the cost of diet, MD and other healthy dietary patterns have been observed to be more expensive than other less healthy choices [14,15,16,17,18]. This may explain, in part, the observed association between low socioeconomic position and adherence to less healthy dietary patterns, which increases the risk of morbidity [19,20,21,22,23,24].

These socioeconomic disparities in diet also affect children and adolescents. Thus, different studies have shown that the intake of fruits, vegetables, and dairy is higher when the socioeconomic status of the parents is more favorable, while the consumption of sweetened beverages and salty foods with high energy density is higher when the socioeconomic conditions are worse [25,26,27,28,29]. However, although it is generally accepted that a low socioeconomic level correlates with lower diet quality, the data come from studies that used different methods to assess the quality of the diet [30].

To our best knowledge, only one study has been conducted in Spanish children to assess the relationship between the cost of diet and adherence to MD. The data from this study were obtained from a cross-sectional survey of the 2–24-year-old Spanish population between 1998 and 2000. The monetary cost of the diet was calculated from the food prices reported in 2000 by the official database of the Spanish Ministry of Economy and Competitiveness, and adherence to the MD was measured by means of the KIDMED index [31]. Since this study did not focus exclusively on children, and it was based on a sample collected between 1998 and 2000, this knowledge needs to be updated among Spanish children.

The aim of this study was to assess the association between the daily cost of the diet and the overall quality of the diet in a cohort of 6–12-year-old Spanish children.

## 2. Materials and Methods

### 2.1. Design and Subjects

A cross-sectional survey was conducted on an initial cohort of 150 children from 6–12-year-old schoolchildren in primary education in the province of Avila (central Spain), during the 2018–2019 academic year. Of these 150 schoolchildren, 15 did not submit informed consent and 5 delivered an incomplete questionnaire, so it was decided to exclude them from the study. Thus, the final cohort consisted of 130 children (47% female). The children were selected at random in two schools, one public and one private, both located in different areas of the province where families of medium socioeconomic status live. The following exclusion criteria were applied: serious illness that influences nutritional status; mental limitation of parents and/or children who may make it difficult to complete the questionnaire.

The study protocols followed the Declaration of Helsinki ethical standards and all procedures were approved by the Ethics Committee of the Ávila Province (ref. GASAV 2020/13). All parents were informed of the purpose of the study and informed consent was obtained by each participating child, which was signed by children and parents.

### 2.2. Questionnaire

The questionnaire, anonymous and self-filled, was structured into three parts: (1) Sociodemographic data (course, birth date, sex), physical activity (type and number of hours), frequency with which children ate in fast-food restaurants. (2) Three 24 h recalls administered according to the European Food Safety Authority (EFSA) [32] validated in Spanish children [8,33,34,35], which included three non-consecutive days, two working days and one weekend or holiday. (3) Data on current use of food supplements, origin of the water that child drinks, as well as data on breastfeeding.

Trained researchers arranged an interview with the director of each school in which they were invited to participate in the study. Upon acceptance by the center, parents and/or guardians of children (hereinafter parents) were informed of the study and voluntary nature of their participation. Parents who decided to participate signed the informed consent along with the questionnaire. Parents were also trained to complete the questionnaire, using a photo album of portions for part 2, and were instructed to describe the amount of food and/or home measures (spoons, glasses, etc.). Parents were invited to allow children to actively participate in completing the questionnaire.

Homemade measures were transformed in grams according to the information contained in Spanish Food Composition Tables [36]. The amount of food was expressed in grams and servings. The size of the servings for this population group was considered according to the White Book on Child Nutrition in Spain [37]. Energy intake were assessed by the Valladolid Center for Endocrinology and Clinical Nutrition Research (IENVA) [38], applying the Spanish Food Composition Database (BEDCA] [39] and Spanish Food Composition Tables [36].

### 2.3. Adherence to the Mediterranean Diet and Compliance with Dietary Reference Intake

Adherence to MD was assessed by means of the Mediterranean Adequacy Index (MAI) [12,40]. Foods were classified in 20 groups: the 18 initially established and, additionally, other dairies (i.e., whole yogurt, skimmed yogurt, dairy desserts, and creamy ice creams) and ready or prepared meals (i.e., mayonnaise, mustard, tomato sauce, savory snacks, over, frozen, pizza soups) that were included in the equation as follows:

$$\begin{array}{c} MAI=\;\frac{\%E\;of\;Mediterranean\;Diet\;highly\;characteristic\;foods}{\%E\;of\;Mediterranean\;Diet\;lowly\;characteristic\;foods}=\\ \frac{\%E\;(bread\;+\;whole\;cereals\;+\;pulses\;+\;potatoes\;+\;vegetables\;+\;fresh\;fruit\;+\;nuts\;+\;fish\;+\;olive\;oil\;+\;wine)}{\begin{array}{c} \%E\;(milk\;+\;cheese\;+\;meat\;+\;eggs\;+\;animal\;fats\;and\;seed\;oils\;+\;sweetened\;beverages\;+\;cakes\;and\;cookies\;\\ +\;sugar\;+\;other\;dairies\;+\;ready\;or\;prepared\;meals) \end{array}} \end{array}$$

### 2.4. Monetary Cost of the Diet

Food prices were obtained from the Household Consumption Database of the Spanish Ministry of Agriculture, Fisheries and Food [41]. The average cumulative prices (average price/kg) reported in the period September 2018 to September 2019 were chosen for the Autonomous Community of Castile and Leon to which the province of Avila belongs. The economic cost of the diet was calculated by multiplying the amount in grams of the food consumed by each child by the corresponding price in grams and adding up the total amount for each participant. The total economic cost of the diet was calculated in €/day and in €/1000 kcal/day.

### 2.5. Statistics

Statistical Package for Social Sciences (SPSS v.25 for Windows, IBM Software Group, Chicago, IL, USA) was used to carry out the statistical analysis. A Kolmogorov–Smirnov test was applied to assess the correct distribution of the data. Results were expressed as means ± standard error of the mean (SEM). The different quintiles of the monetary cost of the diet were compared (€/day and €/1000 kcal/day) according to the sex and age (quartiles) of the participants. The statistical significance of the data was assessed by a one-way analysis of variance (ANOVA). A Bonferroni post hoc test was used to make multiple comparisons. The effect of the adherence to the MD on the monetary cost of the diet (€/day and €/1000 kcal/day) was analyzed by a receiver operating characteristic (ROC) curve and area under the curve (AUC), and by a multivariate logistic regression according to monetary cost of the diet (dependent variable) and MD adherence (lower adherence: MAI ≤ mean; increased adherence: MAI > mean) as an independent variable after adjustments for sex (categorical variable), age, and total energy consumption (continuous variables) to control for potential confounding. Differences between working days and holidays were analyzed using Student t-test for related samples. The level of significance was considered at *p* < 0.05 for all statistics.

## 3. Results

Table 1 shows some characteristics of the participants. No differences were registered between boys and girls relative to age, physical activity, fast-food frequency of consumption, food supplement consumption, and kind of consumed water.

The energy content of the schoolchildren’s diet was 1936.90 ± 39.626 kcal/day, with a lower energy intake on the weekend than on a daily basis (1584.89 ± 42.268; 2372.99 ± 58.449, respectively), with this difference being statistically significant (*p* < 0.001).

**Figure 1:**
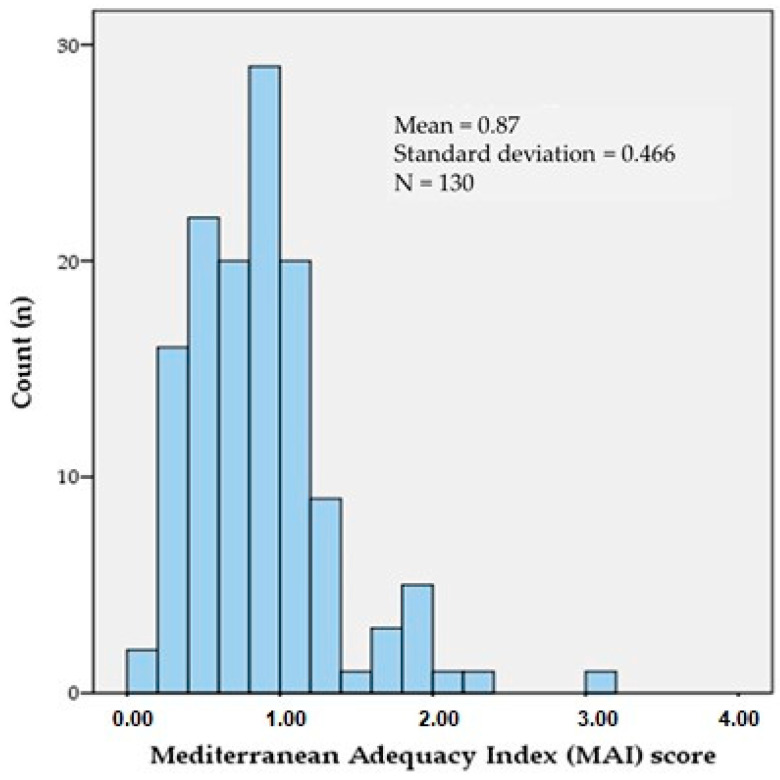
Histogram of the Mediterranean Adequacy Index (MAI) score by schoolchildren.

Figure 1 shows the histogram of MAI score achieved by the schoolchildren cohort. A mean of 0.87 ± 0.466 points was obtained. Around 68.5% of schoolchildren scored less or equal than 1, which would be equivalent to saying that most of the energy ingested came from foods less characteristic of MD. On the other hand, 29.2% of schoolchildren reached a score of 1.01–2, 1.5% scored 2.01–3.00, and in the remaining 0.8%, the score was 3.01–4.00. No significant differences were found between sexes. There were also no statistically significant differences between weekend and daily MAI (*p* = 0.289).

The mean monetary cost of the diet was 3.57 ± 0.679 euros/day. When the cost was standardized to 1000 kcal, a mean of 1.93 ± 0.406 was obtained, with a higher cost in boys (3.68 ± 0.878 €/day and 1.97 ± 0.513 €/1000 kcal/day) than in girls (3.44 ± 0.103 €/day and 1.89 ± 0.642 €/1000 kcal/day), but without significant differences. The cost of diet on public holidays was higher than the daily cost, but without statistically significant differences (*p* (€/day) = 0.780; *p* (€/1000 kcal/day) = 0.780). When the cost of the diet was compared between age quartiles, Bonferroni’s post hoc test showed significant differences, with a maximum diet cost for age quartile 3 (4.069 ± 0.111 €/day and 2.22 €/1000 kcal/day), with differences between age groups (*p* < 0.001) (data not shown).

When sex was considered regarding the distribution of school cohort in the different quintiles of the monetary cost of the diet (Table 2), the highest percentage of girls was found in first quintile of the monetary cost of the diet (31.1% for €/day and €/1000 kcal/day). Among boys, the highest percentage was in the second quintile (29.0%) when the cost was €/day, whereas when that cost was standardized to €/1000 kcal/day, the highest percentage of boys was in the third quintile (26.1%). The differences between sexes within the same quintile were only significant for the third quintile of the monetary cost measured in €/day *(p* = 0.015). Differences between sexes were found when the totality of the monetary cost of the diet was assessed (€/day *p* = 0.002; €/1000 kcal/day *p* = 0.018); data not shown.

The percentage of the budget that schoolchildren earmarked for foods with low MD adherence (62.50 ± 0.95%) was higher than that earmarked for foods with high MD adherence (33.80 ± 1.00%); i.e., 1.05 euros/day more were allocated for the purchase of foods with low MD adherence vs. foods with high MD adherence 2.24 ± 0.59 and 1.20 ± 0.04 €/day, respectively).

**Figure 2:**
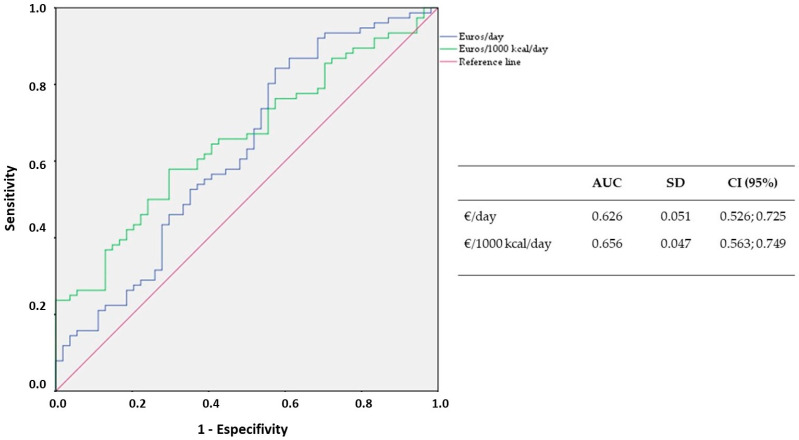
ROC curve of the accuracy of the MD adherence in assessing monetary cost. AUC: Area under the curve.

Figure 2 shows the association between the MD adherence and the monetary cost of the diet using a ROC curve (monetary cost of diet above the mean (€/day > 3.57 and €/1000 kcal/day > 1.93; internal value = 1) vs. monetary cost of diet less than or equal to the mean (€/day ≤3.57 and €/1000 kcal/day ≤ 1.93; internal value = 0)). The area under the curve (AUC) for €/day and €/1000 kcal/day represents 62.6% and 65.6%, respectively. According to AUC values, adherence to MD is a moderate predictor of the monetary cost of the diet.

Table 3 shows the association of monetary cost of the diet and adherence to the Mediterranean diet, by means of a multivariate adjusted logistic regression (B, standard error, *p* (Wald), odds ratio and 95% CI) considering monetary cost of diet lower than mean (€/day ≤ 3.57 and €/1000 kcal/day ≤ 1.93; internal value = 0) as a reference value. After adjusting for possible confounders, considering children with lower adherence to MD (MAI ≤ 0.87) compared to those with higher adherence (MAI > 0.87), a higher cost of diet in schoolchildren was related to above-average MAI values [OR (€/day) = 1.640; 95%CI: 0.682; 3.944], but with no significant differences (*p* = 0.269). When the cost of the diet was standardized to 1000 kcal, the same direct relationship between the cost of the diet and the MAI was observed [OR (€/100 kcal/day) = 3.012; 95%CI: 1.291; 7.026] but showing significant association (*p* = 0.011).

Monetary cost of diet ≤ mean as reference value and monetary cost of diet > mean (dependent variable) and MD adherence (lower adherence: MAI ≤ mean; increased adherence: MAI > mean) as an independent variable after adjustments by sex, school (rural or urban) (categorical variables), age, and total energy consumption (continuous variables). CI: confidence interval.

## 4. Discussion

The main findings of this study are that there is a direct and significative association between MD adherence, as measured by MAI, and the monetary cost of the diet standardized to €/1000 kcal/day. Current results are in line with those obtained in previous studies carried out in 2–24-year-old Spanish population using the KIDMED index [31], and among Portuguese [42] and Swedish children [43,44] based on the KIDMED index and 2005 Healthy Eating Index (HEI), respectively. These previous studies pointed out that a positive and significant relationship was observed with the monetary cost of the diet (€/day and €/1000 kcal/day).

In a previous German children and adolescent cohort study [44], records ranked similarly to this current study, considering high-quality diet scores above median and low-quality scores below median and applying the Nutrient Quality Index (NQI) and the Healthy Nutrition Score for Kids and Youth (HuSKY). These authors reported no significant association in low-quality records, but the association was direct and significant in high-quality records [44]. In the current study, similar results were obtained because no significant association was found when the cost of the diet was analyzed at €/day, but the association was significant when the cost was standardized to €/1000 kcal/day, showing that assessment using absolute values (€/day) could mask the results and standardization is compulsive in this analysis. Furthermore, the availability of money that children have for their own expenses could also influence the results. Accordingly, a study conducted in 2019 on Northeastern Spanish children and adolescents found a negative association between the availability of money that the studied subjects had and adherence to MD [45].

All schoolchildren participating in the current study scored MAI below 5, which means low adherence to MD. Studies on adherence to MD previously conducted in children agree with those obtained in the current study. Thus, in 2014, most of children and adolescents in the Mediterranean region showed low adherence to the MD, increasing the intake of processed foods and saturated fats [46]. Similar conclusions were obtained in 2013 among rural and urban adolescents in Southern Spain [47], showing that most of the subjects had low–medium adherence to MD. In a 2009 study, most Cyprus children showed low adherence to MD [48]. In 2018, Northern Italian primary and secondary children and adolescents showed that poor adherence to MD was more prevalent in primary schools than in high schools [49], but Northeastern Spanish children showed worse adherence to MD among secondary schoolers [45]. Other previous studies found moderate–high adherence to MD in children and adolescents [31,42,43,50,51]. The adherence to MD was not different between boys and girls in the current study. Although there may be some mismatch previously [52]; most studies on the degree of adherence to MD in this population group did not show differences between sexes, except for slight variations [53].

The results of the current study show that the mean monetary cost of the diet was 3.57 ± 0.679 euros, and when the cost was standardized to €/1000 kcal/day, a mean of 1.93 ± 0.406 was obtained. These results are like those obtained in previous studies [31,42]. The monetary cost of the diet was higher in boys than in girls, results that match those obtained in the study carried out in the 2–24-year-old Spanish population [31]. Therefore, the current results show that studied children from Central Spain showed similar adherence to MD among other Mediterranean children. The reason why these Mediterranean children showed low adherence will need further assessment. Among the widest variety of foods present in the healthiest dietary patterns, some MD key foods, such as fruits, vegetables, and fish as well as healthier options like lean meats and low-fat products are usually associated with higher costs [15,42,43,54]. In the schoolchildren participating in the current study, consumption of fruits, vegetables, fish, and lean meats were much lower than the recommendations, which are in line with the low adherence to MD using a low part of their budget to the consumption of these foods.

When analyzing MD from a cost perspective, it is important to note that not all foods with high nutrient density have a high cost; so, it is possible to design a diet following the Mediterranean dietary pattern using the lowest monetary cost options in each food group. Accordingly, it is important to point out that in the area in which the current study has been developed, the proximity market is common, and many families have their own orchards; so, it would be possible to follow up on a healthier dietary pattern than currently observed. Therefore, it will be desirable to design nutrition education programs that insist on these aspects. Future research is needed to obtain greater validation of MD indexes in terms of consistency and reproducibility. Cohort and intervention studies should assess further MD associations with behavioral and health factors.

## 5. Strengths and Limitations

The current study provides insights into the monetary costs of the diet and its association with adherence to MD, an issue that is becoming relevant in public health worldwide due to the current economic crisis in many Mediterranean countries along with the need to design nutrition education programs that integrate healthy diet designs with more cost-effective options.

The main limitation of the current study is that the low adherence to MD found in schoolchildren did not allow benchmarking on the cost of the diet for wider ranges of MAI scores. However, the categorization of the sample in two groups according to the MAI score obtained has allowed a real analysis of the schoolchildren. Another limitation was the validity and reliability of dietary consumption data from surveys, being well known for the potential for bias [55,56]. In studies aimed at children, this becomes more relevant as they have a greater difficulty in estimating the amount of consumed food, and in the case of surveys being conducted on parents, they have a limited ability to assess all eaten foods outside the family environment. There is evidence that the combined use of different methods of evaluating dietary intake (quantitative and non-quantitative questionnaires) with biomarkers, and integrated with statistical models, can give more accurate estimates of the usual individual intake [57,58]. The current study used a journal or dietary record used and validated in Spanish children [8,33,34,35]. In addition, parents were previously trained and quality control was followed to minimize bias. Finally, the design of the study is cross-sectional, providing evidence of association, but not causal relationships.

## 6. Conclusions

In a cohort of Spanish schoolchildren with low adherence to the Mediterranean diet, a higher cost of the diet standardized to 1000 kcal was associated with above-average MAI values.

## Figures and Tables

**Table 1:** Demographic, lifestyle, and food allergies or intolerances among studied children.

	Total (*n* = 130)	Boys (*n* = 69)	Girls (*n* = 61)	*p*
Age (years: mean ± SEM	9.51 ± 0.159	9.54 ± 0.226	9.48 ± 0.224	0.849
Physical activity (hours/week): mean ± SEM	5.53 ± 0.225	5.58 ± 0.272	5.47 ± 0.370	0.804
Fast-food restaurants (days/month): mean ± SEM	1.39 ± 0.132	1.29 ± 0.150	1.51 ± 0.225	0.412
Food supplements: *n* (%)	6 (4.6)	2 (2.9)	4 (6.6)	0.321
Kind of water:				0.351
− Faucet: *n* (%)	30 (23.1)	18 (26.1)	12 (19.7)	
− Bottled: *n* (%)	88 (67.7)	43 (62.3)	45 (73.8)	
− Other: *n* (%)	12 (9.2)	8 (11.6)	4 (6.6)	
Breastfeeding: *n* (%)	114 (87.7)	58 (84.1)	56 (91.8)	0.18

Differences between sex were analyzed by one-way ANOVA or Pearson chi-square for continuous and categorical variables, respectively.

**Table 2:** Distribution of schoolchildren across quintiles of daily monetary diet cost (€/day and €/1000 kcal/day).

		Q1	Q2	Q3	Q4	Q5
€/day	Total	2.49 (2.37–2.62)	3.13 (3.08–3.17)	3.55 (3.50–3.60)	4.03 (3.97–4.10)	4.64 (4.50–4.78)
	Boys	7 (10.1%)	20 (29.0%)	10 (14.5%)	15 (21.7%)	17 (24.6%)
		2.54 (2.27–2.81)	3.12 (3.07–3.18)	3.48 (3.40–3.56)	4.06(3.98–4.14)	4.61(4.40–4.81)
	Girls	19 (31.1%)	6 (9.8%)	16 (26.2%)	11 (18.0%)	9 (14.8%)
		2.47 (2.32–2.63)	3.13 (3.02–3.25)	3.60 (3.54–3.66)	4.00 (3.90–4.11)	4.69 (4.51–4.87)
	*p*	0.636	0.856	0.015	0.369	0.572
€/1000 kcal/day	Total	1.32 (1.24–1.40)	1.66 (1.61–1.70)	1.92 (1.89–1.95)	2.15 (2.11–2.19)	2.59 (2.49–2.70)
	Boys	7 (10.1%)	16 (23.2%)	18 (26.1%)	16 (23.2%)	12 (17.4%)
		1.27 (1.14–1.39)	1.64 (1.58–1.70)	1.93 (1.89–1.97)	2.17 (2.11–2.22)	2.62 (2.45–2.80)
	Girls	19 (31.1%)	10 (16.4%)	8 (13.1%)	10 (16.4%)	14 (23.0%)
		1.34 (1.24–1.45)	1.69 (1.62–1.75)	1.92 (1.90–1.94)	2.13 (2.05–2.20)	2.57 (2.42–2.72)
	*p*	0.398	0.311	0.688	0.381	0.607

The data are expressed in *n* (% within sex) and average (95%CI). The differences between the sexes for the stockings were analyzed by one-way ANOVA.

**Table 3:** Association of monetary cost of diet and adherence to the Mediterranean diet.

	B	Standard Error	*p* (Wald)	OR (Exp(B))	95%CI
€/day	0.495	0.448	0.269	1.640	0.682; 3.944
€/1000 kcal/day	1.103	0.432	0.011	3.012	1.291; 7.026

## Data Availability

There are restrictions on the availability of data for this trial, due to the signed consent agreements around data sharing, which only allow access to external researchers for studies following the project purposes. Requestors wishing to access the trial data used in this study can make a request to pep.tur@uib.es.

## References

[1] World Heatlh Organization (2003). Diet, Nutrition and the Prevention of Chronic Diseases. https://apps.who.int/iris/bitstream/handle/10665/42665/WHO_TRS_916.pdf;jsessionid=0CBB7BE4EEFE0B8A6BE84BB4FB47B691?sequence=1.

[2] O’Neil A., Quirk S.E., Housden S., Brennan S.L., Williams L.J., Pasco J.A., Berk M., Jacka F.N. (2014). Relationship Between Diet and Mental Health in Children and Adolescents: A Systematic Review. Am. J. Public Health.

[3] Martínez-González M.A., Salas-Salvadó J., Estruch R., Corella D., Fitó M., Ros E. (2015). Benefits of the Mediterranean Diet: Insights From the PREDIMED Study. Prog. Cardiovasc. Dis..

[4] Sofi F., Abbate R., Gensini G.F., Casini A. (2010). Accruing evidence on benefits of adherence to the Mediterranean diet on health: An updated systematic review and meta-analysis. Am. J. Clin. Nutr..

[5] Schröder H. (2007). Protective mechanisms of the Mediterranean diet in obesity and type 2 diabetes. J. Nutr. Biochem..

[6] Estruch R., Ros E., Salas-Salvadó J., Covas M.-I., Corella D., Arós F., Gómez-Gracia E., Ruiz-Gutiérrez V., Fiol M., Lapetra J. (2018). Primary Prevention of Cardiovascular Disease with a Mediterranean Diet Supplemented with Extra-Virgin Olive Oil or Nuts. N. Engl. J. Med..

[7] Giannini C., Diesse L., D´Adamo E., Chiavaroli V., de Giorgis V., Di Lorio C., Chiarelli F., Mohn A. (2014). Influence of the Mediterranean Diet on Carotid Intima-Media Thickness in Hypercholesterolaemic Children: A 12-month Intervention Study. Nutr. Metab. Cardiovasc. Dis..

[8] Ministerio de Sanidad, Consumo y Binestar Social. Agencia Española de Consumo, Seguridad Alimentaria y Nutrición Encuesta ENALIA. Encuesta Nacional de Alimentación en la Población Infantil y Adolescente. http://www.aecosan.msssi.gob.es/eu/AECOSAN/web/seguridad_alimentaria/subdetalle/enalia.htm.

[9] Tur J.A., Romaguera D., Pons A. (2005). The Diet Quality Index-International (DQI-I): Is it a useful tool to evaluate the quality of the Mediterranean diet?. Br. J. Nutr..

[10] Trichopoulou A., Kouris-Blazos A., Wahlqvist M.L., Gnardellis C., Lagiou P., Polychronopoulos E., Vassilakou T., Lipworth L., Trichopoulos D. (1995). Diet and overall survival in elderly people. BMJ.

[11] D´Alessandro A., De Pergola G. (2015). Mediterranean Diet and Cardiovascular Disease: A Critical Evaluation of a Priori Dietary Indexes. Nutrients.

[12] Fidanza F., Alberti A., Fruttini D. (2005). The Nicoreta Diet: The Reference Italian Mediterranean Diet. World Rev. Nutr. Diet.

[13] French S.A. (2003). Pricing Effects on Food Choices. J. Nutr..

[14] Schröder H., Marrugat J., Covas M.I. (2006). High monetary costs of dietary patterns associated with lower body mass index: A population-based study. Int. J. Obes..

[15] Lopez C.N., A Martinez-Gonzalez M., Sanchez-Villegas A., Alonso A., Pimenta A.M., Bes-Rastrollo M. (2009). Costs of Mediterranean and western dietary patterns in a Spanish cohort and their relationship with prospective weight change. J. Epidemiol. Community Health.

[16] Schröder H., Serra-Majen L., Subirana I., Izquierdo-Pulido M., Fitó M., Elosua R. (2016). Association of increased monetary cost of dietary intake, diet quality and weight management in Spanish adults. Br. J. Nutr..

[17] Rao M., Afshin A., Singh G., Mozaffarian D. (2013). Do healthier foods and diet patterns cost more than less healthy options? A systematic review and meta-analysis. BMJ Open.

[18] Afshin A., Peñalvo J., Del Gobbo L., Silva J., Michaelson M., O´Flaherty M., Capewell S., Spiegelman D., Danaei G., Mozaffarian D. (2017). The prospective impact of food pricing on improving dietary consumption: A systematic review and meta-analysis. PLoS ONE.

[19] Méjean C., Droomers M., Van Der Schouw Y.T., Sluijs I., Czernichow S., Grobbee D.E., Bueno-De-Mesquita H.B., Beulens J.W.J. (2013). The contribution of diet and lifestyle to socioeconomic inequalities in cardiovascular morbidity and mortality. Int. J. Cardiol..

[20] Laaksonen M., Talala K., Martelin T., Rahkonen O., Roos E., Helakorpi S., Laatikainen T., Prättälä R. (2007). Health behaviours as explanations for educational level differences in cardiovascular and all-cause mortality: A follow-up of 60,000 men and women over 23 years. Eur. J. Public Health.

[21] Stringhini S., Sabia S., Shipley M., Brunner E., Nabi H., Kivimaki M., Singh-Manoux A. (2010). Association of socioeconomic position with health behaviors and mortality. JAMA.

[22] Giskes K., Avendano M., Brug J., Kinst A.E. (2010). A systematic review of studies on socioeconomic inequalities in dietary intakes associated with weight gain and overweight/obesity conducted among European adults. Obes. Rev..

[23] Darmon N., Drewnowski A. (2008). Does social class predict diet quality?. Am. J. Clin. Nutr..

[24] De Irala-Estévez J., Groth M., Johansson L., Oltersdorf U., Prättälä R., A Martínez-González M. (2000). A systematic review of socio-economic differences in food habits in Europe: Consumption of fruit and vegetables. Eur. J. Clin. Nutr..

[25] Grosso G., Marventano S., Nolfo F., Rametta S., Bandini L., Ferranti R., Bonomo M.C., Matalone M., Galvano F., Mistretta A. (2013). Personal Eating, Lifestyle, and Family-Related Behaviors Correlate with Fruit and Vegetable Consumption in Adolescents Living in Sicily, Southern Italy. Int. J. Vitam. Nutr. Res..

[26] Lehto E., Ray C., Velde S.T., Petrova S., Duleva V., Krawinkel M., Behrendt I., Papadaki A., Kristjansdottir A., Thorsdottir I. (2014). Mediation of parental educational level on fruit and vegetable intake among schoolchildren in ten European countries. Public Health Nutr..

[27] Desbouys L., De Ridder K., Rouche M., Castetbon K. (2019). Food Consumption in Adolescents and Young Adults: Age-Specific Socio-Economic and Cultural Disparities (Belgian Food Consumption Survey 2014). Nutriens.

[28] Di Noia J., Byrd-Bredbenner C. (2014). Determinants of fruit and vegetable intake in low-income children and adolescents. Nutr. Rev..

[29] Drewnoswski A., Rhem C. (2015). Socioeconomic gradient in consumption of whole fruit and 100% fruit juice among US children and adults. Nutr. J..

[30] Nikolić M., Glibetić M., Gurinović M., Milešević J., Khokhar S., Chillo S., Abaravicius J.A., Bordoni A., Capozzi F. (2014). Identifying critical nutrient intake in groups at risk of poverty in Europe: The CHANCE project approach. Nutrients.

[31] Schröder H., Gomez S.F., Ribas-Barba L., Pérez-Rodrigo C., Bawaked R.A., Fíto M., Serra-Majem L. (2016). Monetary Diet Cost, Diet Quality, and Parental Socioeconomic Status in Spanish Youth. PLoS ONE.

[32] European Food Safety Authoruty (2014). Guidance on the EU Menu Methodology. EFSA J..

[33] Leis R., Rojo T. (2004). Cardiovascular risk factors among obese children and adolescents. The Galinut Study. JPGN..

[34] Leis R., Pavón P., Queiro T., Recarey D., Tojo R. (1999). Atherogenic diet and blood lipid profile in children and adolescents from Galicia, NW Spain. The Galinut Study. Acta Paediatr..

[35] Subdirección General de Higiene de Los Alimentos (1994). Consumo de Alimentos y Estado Nutricional de la Población Escolar de la Comunidad Autónoma de Madrid.

[36] Moreiras O., Carbajal A., Cabrera L., Cuadrado C. (2016). Tablas de Composición de Alimentos. Guía de Practices.

[37] Pastor R., Rivero M., Moreno L., Dalmau J., Moreno J., Aliaga A., García A., Varela G., Ávila J.M. (2015). Programación de menús infantiles. Libro Blanco de la Nutrición Infantil en España.

[38] Centro de Investigación de Endocrinología y Nutrición Clínica (2010). Calculadora de Dietas. http://www.ienva.org/CalcDieta/.

[39] Ministerio de Sanidad, Servicios Sociales e Igualdad. Ministerio de Ciencia e Innovación Base de Datos Española de Composición de Alimentos (BEDCA). http://www.bedca.net/bdpub/index.php.

[40] Alberti-Fidanza A., Fidanza F. (2004). Mediterranean Adequacy Index of Italian diets. Public Health Nutr..

[41] Ministerio de Agricultura, Pesca y Alimentación Base de Datos de Consumo en Hogares. https://www.mapa.gob.es/app/consumo-en-hogares/consulta11.asp.

[42] Albuquerque G., Moreira P., Rosário R., Araújo A., Teixeira V.H., Lopes O., Padrão P. (2017). Adherence to the Mediterranean diet in children: Is it associated with economic cost?. Porto Biomed. J..

[43] Rydén P., Hagfors L. (2011). Diet cost, diet quality and socio-economic position: How are they related and what contributes to differences in diet costs?. Public Health Nutr..

[44] Alexy U., Schwager V., Kersting M. (2014). Diet quality and diet costs in German children and adolescents. Eur. J. Clin. Nutr..

[45] Arcila-Agudelo A.M., Ferrer-Svoboda C., Torres-Fernàndez T., Farran-Codina A. (2019). Determinants of Adherence to Healthy Eating Patterns in a Population of Children and Adolescents: Evidence on the Mediterranean Diet in the City of Mataró (Catalonia, Spain). Nutrients.

[46] Naska A., Trichopoulou A. (2014). Back to the future: The Mediterranean diet paradigm. Nutr. Metab. Cardiovasc. Dis..

[47] Grao-Cruces A., Nuviala A., Fernández- Martínez A., Porcel-Gálvez A., Moral-García J., Martínez-López E. (2013). Adherencia a la dieta mediteránea en adolescentes rurales y urbanos del sur de España, satisfacción con la vida, antropometría y actividades físicas y sedentarias. Nutr. Hosp..

[48] Lazarou C., Panagiotakos D.B., Matalas A.-L. (2009). Level of adherence to the Mediterranean diet among children from Cyprus: The CYKIDS study. Public Health Nutr..

[49] Archero F., Ricotti R., Solito A., Carrera D., Civello F., Di Bella R., Bellone S., Prodam F. (2018). Adherence to the Mediterranean Diet among School Children and Adolescents Living in Northern Italy and Unhealthy Food Behaviors Associated to Overweight. Nutrients.

[50] Metro D., Tardugno R., Papa M., Bisignano C., Manasseri L., Calabrese G., Gervasi T., Dugo G., Cicero N. (2018). Adherence to the Mediterranean diet in a Sicilian student population. Nat. Prod. Res..

[51] Peng W., Goldsmith R., Berry E.M. (2016). Demographic and lifestyle factors associated with adherence to the Mediterranean diet in relation to overweight/obesity among Israeli adolescents: Findings from the Mabat Israeli national youth health and nutrition survey. Public Health Nutr..

[52] Zapico A., Blández J., Fernández E. (2010). Sobrepeso, obesidad y adecuación a la dieta mediterránea en adolescentes de la Comunidad de Madrid. Arch. Med. Deporte.

[53] Kontogianni M.D., Vidra N., Farmaki A.-E., Koinaki S., Belogianni K., Sofrona S., Magkanari F., Yannakoulia M. (2008). Adherence Rates to the Mediterranean Diet Are Low in a Representative Sample of Greek Children and Adolescents. J. Nutr..

[54] Darmon N., Briend A., Drewnowski A. (2004). Energy-dense diets are associated with lower diet costs: A community study of French adults. Public Health Nutr..

[55] Arija V., Salas-Salvadó J., Bonada A., Trallero R., Saló M., Burgos R. (2014). Métodos de valoración del consumo alimentario. Nutrición y Dietética Clínica.

[56] Arija V., Abellana R., Ribot B., Ramón J.M. (2015). Biases and adjustments in nutritional assessments from dietary questionnaires. Nutr. Hosp..

[57] Falomir Z., Arregui M., Madueño F., Corella D. (2012). Coltell, Óscar Automation of Food Questionnaires in Medical Studies: A state-of-the-art review and future prospects. Comput. Biol. Med..

[58] Illner A.-K., Freisling H., Boeing H., Huybrechts I., Crispim S.P., Slimani N. (2012). Review and evaluation of innovative technologies for measuring diet in nutritional epidemiology. Int. J. Epidemiol..

